# Shortening the Journey to Cure: A Quasi-experimental Trial Comparing Seven-Day and 14-Day Primaquine Radical Therapy for Vivax Malaria

**DOI:** 10.7759/cureus.89946

**Published:** 2025-08-12

**Authors:** Khalid Shahab, Abdul Salam, Noman Salih, Muhammad Bilal, Mehmood Jan

**Affiliations:** 1 Medicine, Khyber Girls Medical College, Peshawar, PAK; 2 Internal Medicine, Hayatabad Medical Complex Peshawar, Peshawar, PAK; 3 Internal Medicine, Khyber Medical College, Peshawar, PAK

**Keywords:** 7-day regimen, malaria, plasmodium vivax, primaquine, radical therapy

## Abstract

Introduction

Because dormant liver-stage hypnozoites can cause recurrence, Plasmodium vivax (P. vivax) malaria poses serious problems. Although the standard treatment for radical cure is 14 days of primaquine medication, this lengthy course of treatment frequently causes poor adherence. In contrast to the typical 14-day low-dose regimen, this study assesses the effectiveness, safety, and adherence of a condensed seven-day high-dose primaquine regimen.

Objectives

The objectives of this study are to assess and compare the efficacy of seven-day high-dose primaquine therapy with 14-day low-dose therapy.

Methodology

Over nine months, this quasi-experimental study was carried out at the Hayatabad Medical Complex's General Medicine Unit in Peshawar, Pakistan. One hundred patients with confirmed P. vivax malaria, ages 18 to 76, were split into two groups. Group A (intervention group; n=50) was given primaquine (1 mg/kg/day) for seven days, whereas Group B (control group; n=50) was given primaquine (0.5 mg/kg/day) for 14 days. Assessments were conducted again on days 14, 28, 60, and 90 to look for relapse of febrile illness and/or parasitemia. IBM SPSS Statistics for Windows, Version 23 (Released 2015; IBM Corp., Armonk, New York, United States) was used to analyse treatment outcomes, such as adverse events, adherence, and illness recurrence.

Results

The disease recurrence rate was substantially lower in Group A (2%), which was receiving a shorter course of therapy, as compared to Group B (26%), which was given 14-day therapy. Chi-square analyses show significance with a p-value of 0.012. Additionally, the shorter regimen had a higher adherence rate (94%) compared to the 14-day regimen (76%) (p = 0.012). Both regimens were well tolerated, with a slightly increased occurrence of very mild gastrointestinal symptoms in Group A. No serious side effects were noted.

Conclusion

Similar efficacy to the conventional 14-day therapy is provided by the seven-day high-dose primaquine regimen, which also has an acceptable safety profile and much-improved adherence. In areas with limited resources where adherence to prolonged therapy is difficult, this condensed regimen may improve treatment outcomes.

## Introduction

Malaria remains a significant global health problem, particularly in resource-limited areas, with Plasmodium vivax (P. vivax) being responsible for the majority of cases in endemic regions. P. vivax has the widest geographic range of any malaria parasite, with Africa and Asia being the major contributors [1]. It has been estimated that around four million malaria cases were reported in 2023 in Southeast Asia, and 48% were due to P. vivax [2]. Accounting for disease burden, P. vivax poses challenges due to its ability to cause recurrence through dormant liver-stage hypnozoites [3,4]. Multiple studies suggest that a relapse due to hypnozoites rather than reinfection is largely responsible for disease recurrence in patients suffering from P. vivax malaria [5]. Strains of P. vivax from different geographic areas have been shown to have a range of relapse periodicities, with tropical strains relapsing more quickly and often than temperate strains [3]. Furthermore, most P. vivax relapses occur in the first three months of the initial insult, while long-term relapses are relatively rare [6].

Different treatments are in practice, including artemether-based combination therapy or chloroquine, paired with primaquine. Primaquine, an 8-aminoquinoline, is the only FDA-approved drug currently available for the radical cure of vivax malaria, targeting both the blood-stage gametocytes and dormant liver-stage hypnozoites. However, conventional 14-day primaquine radical therapy is often associated with poor adherence, particularly in resource-constrained settings where extended treatment durations are impractical for many patients [7]. This poor adherence is one of the primary barriers to its effectiveness, as the cumulative dose of primaquine determines its efficacy.

In his non-inferiority trial, Taylor et al. demonstrate how a shorter, higher-dose regimen may be able to address adherence problems without sacrificing therapeutic results [6]. By reducing the treatment duration to seven days while maintaining an equivalent cumulative dose, such a regimen aims to enhance patient compliance and treatment success. This approach is particularly relevant in endemic regions where socioeconomic factors and healthcare access influence patient adherence [8]. However, high-dose therapy has been associated with an increased risk of causing haemolysis in G6PD-deficient patients, methaemoglobinaemia, gastrointestinal (GI) discomfort, etc. This is particularly challenging as the geographic distribution of G6PD deficiency, the most common inherited human enzymopathy [9], mirrors that of malaria because it provides some protection against the disease [9,10]. This experimental trial was designed to compare the efficacy, adherence, and safety profiles of seven-day and 14-day primaquine regimens for vivax malaria.

## Materials and methods

A quasi-experimental model was designed to compare the two regimes among patients at Hayatabad Medical Complex, a tertiary care hospital in the Peshawar district, Khyber Pakhtunkhwa, Pakistan. The study was initiated after obtaining written approval from the Institutional Research & Ethical Board, reference no. 2230. Data was gathered over nine months from March 2024 to December 2024. A convenient non-probability sampling method was used to enroll 100 patients fulfilling the inclusion and exclusion criteria. Patients diagnosed with P. vivax, either by fluorescence microscopy or Leishman-stained blood smear, were included in the study. This study was retrospectively registered with the Australian New Zealand Clinical Trials Registry (ANZCTR) under the registration number (ACTRN12625000524493).

Participants were assigned to two groups in an alternating fashion, with every other patient placed into Group A and the next into Group B, ensuring equal distribution (50 patients in each group). Group A (intervention group) was given primaquine at a dose of 1 mg/kg/day for seven days, while Group B (control group) was given primaquine at a dose of 0.5 mg/kg/day for 14 days. Daily doses were taken in an equally divided BD regimen. A single batch of 15 mg tablets of primaquine was used in all patients to minimise confounders.

Data were collected regarding patient demographics (age, gender, body weight, medical history), drug regimen, and disease course (duration & dose of treatment, compliance, disease relapse, side effects). Follow-up visits were conducted on days 14, 28, 60, and 90. During follow-up, fluorescence microscopy and smear staining with Leishman stain were repeated to detect parasitemia with P. vivax gametocytes, as these methods are cost-effective. For adherence, a verbal interview from the patient and the pill count method were used. In addition, inflammatory markers and complete blood counts were conducted. Treatment success, assessed as a composite primary outcome, was defined as the absence of both clinical relapse (no recurrence of febrile illness) and parasitological relapse (no detection of P. vivax gametocytes on blood film during follow-up). Recurrence of febrile illness and parasitemia with P. vivax gametocytes in blood film during follow-up was considered a disease relapse.

Inclusion criteria

Patients aged 18 years and above, including both genders, diagnosed with P. vivax malaria confirmed by peripheral smear examination/malaria fluorescent test, were included in the study.

Exclusion criteria

The study excluded pregnant or lactating mothers, patients with G6PD deficiency, bleeding diathesis or haemolytic anaemia, severe complicated malaria, hypersensitivity to drugs, those using drugs that could interact with primaquine, and those who had recently received a blood transfusion (in the previous 90 days).

Statistical analyses

The data was compiled in an Excel sheet and analysed using IBM SPSS Statistics for Windows, Version 23 (Released 2015; IBM Corp., Armonk, New York, United States). Both groups were compared for treatment adherence, disease recurrence, and side effects using the chi-square test. A p-value of less than 0.05 was considered statistically significant.

## Results

This study included 100 participants satisfying the selection criteria, evenly distributed between Group A (intervention group) (n=50), which received a seven-day regimen, and Group B (control group) (n=50), which received a 14-day regimen. No dropouts were there in this study. The demographic details of each group are presented in Table 1.

**Table 1:** Demographic characteristics of patients in the two study groups. "+/-": The value after (+/-) denotes the 1 S.D. (standard deviation) value, i.e., elaborating the spread of data around the mean value.

Characteristics	Group A (Seven-Day Regime)	Group B (14-Day Regime)
Number of patients included in the study	50 (100%)	50 (100%)
Number of patients who completed the study	47 (94%)	38 (76%)
Mean age (years)	30.36 +/- 13.3	32.08 +/- 14.7
Gender		
Female	29 participants (58%)	17 participants (34%)
Male	21 participants (42%)	33 participants (66%)
Body weight (kg)	70.74 +/- 10.06	69.72 +/- 11.79
Baseline haemoglobin (gm/dl)	12.9 +/- 0.9	12.8 +/- 0.9

The mean age of participants was 31.2 years (range: 18-76 years). The gender distribution was 46% (n=46) female and 54% (n=54) male. Both groups were comparable in baseline characteristics, i.e., body weight, haemoglobin levels, etc. (Table 1), and confounders were sufficiently minimised.

**Figure 1:**
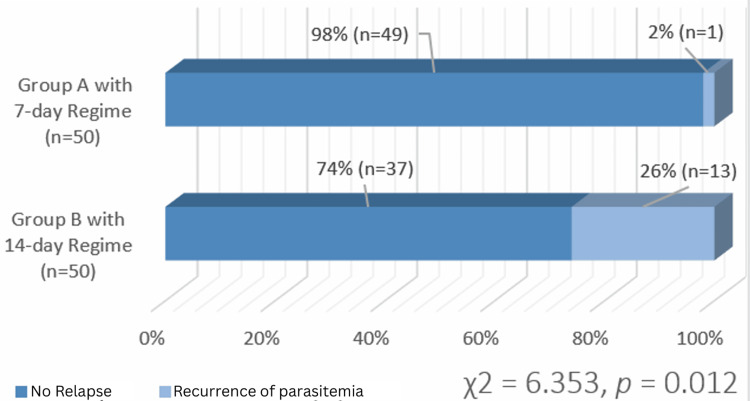
Comparison of disease recurrence between Group A and Group B and their chi-square analyses with p-value. Group A (primaquine at a dose of 1 mg/kg/day for a total of seven days) Group B (primaquine at a dose of 0.5 mg/kg/day for a total of 14 days)

**Figure 2:**
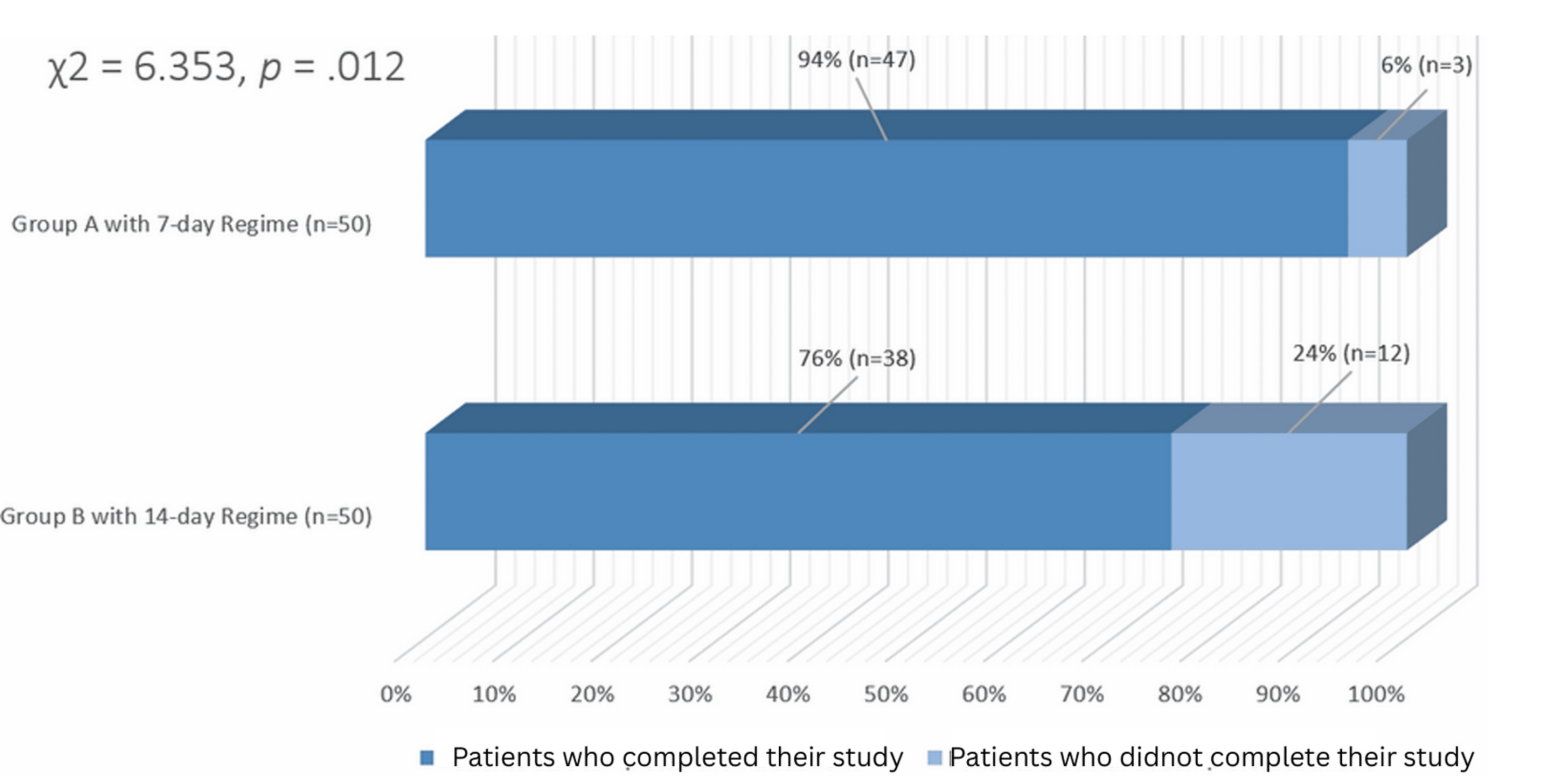
Comparison of treatment compliance between Group A and Group B, with their chi-square test and p-value. Group A (primaquine at a dose of 1 mg/kg/day for a total of 7 days) Group B (primaquine at a dose of 0.5 mg/kg/day for a total of 14 days)

As shown in Figure 1, the recurrence of febrile symptoms and parasitemia was significantly lower in Group A (2%, n = 1) than in Group B (26%, n = 13), supported by chi-square analysis χ² (1) = 6.353, p = 0.012. Additionally, Figure 2 demonstrates that adherence was significantly higher in Group A (94%, n = 47) compared to Group B (76%, n = 38), having a chi-square analysis i.e., χ²(1) = 6.353 with a p-value of 0.012. However, the relapse of disease among the patients adherent to the treatment was not significantly different i.e. Group A: 47 adherent patients → 1 relapse (2.1%) and Group B: 38 adherent patients → 2 relapses (5.3%); 12 non-adherent → 11 relapses (91.7%).

It emphasises that the increased efficacy of the shorter regimen is because of more treatment adherence. Mild GI discomfort was reported in both Group A (n=4, 8%) and Group B (n=3, 6%), with no statistically significant difference.

An analysis of treatment non-compliance patterns indicated that participants in Group B cited the length of the regimen as the cause for discontinuation, while logistic challenges such as access to follow-up were noted in Group A. This suggests that shorter regimens may more effectively address barriers to adherence.

## Discussion

P. vivax malaria is a significant problem in tropical areas because it can relapse due to the existence of dormant hypnozoites. The established treatment that eradicates hypnozoites is primaquine administration as an 8-aminoquinoline. However, the optimal dosage and treatment period remain under investigation to date.

As early as the 1970s, research into anti-relapse treatment with primaquine has been going on, which is alarming because it includes some side effects such as haemolysis and methemoglobinemia, which require further attention. In addition, treatment strategies and designs of clinical trials have been the focus of study. Chu et al. reported in their study that fewer days of high doses of primaquine, for example, a week, are as effective as the two-week standard course and are better tolerated, although the risks of high doses, like methemoglobinemia and GI effects, are slightly increased [8].

Local context and demographics

For this study, we received 100 respondents from Peshawar, Pakistan, with an average age of 31.2 years (range 18 to 76 years), of which 54 percent were male and 46 percent were female. This is contrary to a study on the Asian population that found that men were more protected against vivax malariae because of the haemolytic effect of G6PD, its X-linked genetics, and a positive selection of its variant [10]. Numerous socioeconomic and biological factors can be used to explain the male-to-female ratios and distribution of malaria. For example, men in this region are more likely than women to engage in outdoor activities and wear less-covered clothing. This may help to explain why there were more men in our study because they are more likely to get bitten by mosquitoes.

Effectiveness of the treatment methodologies

We gathered statistics indicating that Group A, which was subjected to primaquine for seven days, is far better than Group B, which was administered low doses for the long term, i.e., for 14 days. For example, only two percent of the patients in Group A relapse, whereas Group B had a 26% relapse rate. This comes out the same as other studies consulted in the course of this paper. Indeed, Chu et al. proved that when higher doses are used, shorter regimens not only improve patient adherence but are also overall more effective [8]. However, it is also important to note that the bulk of occurrences of disease relapses come from those patients who did not stick to therapy. In this study, we can see that compliant patients, be they with shorter or longer treatment plans, do not relapse. We were able to observe a change in compliance within the two groups whereby Group A had compliance of ninety-four percent and Group B had compliance of seventy-six percent (see Figure 2).

In this aspect, non-compliance was coupled with the amount of time the patient was subjected to as a treatment client. Where these geographical or societal gaps incapacitate the ability of patients to serve long treatment, these shorter doses of primaquine are easy to tolerate and thus more effective.

Thus, to summarise, both regimens had equal efficacy upon completion; however, in practical life situations, the shorter regimen, which is followed more, will have the most beneficial results.

Safety profile

Our study did not face significant issues related to safety, and some patients reported mild GI symptoms in both treatment groups.

Group A patients encountered these side effects more often (8%) than Group B (6%). The results are supported by Taylor et al. [6]. One should note that no serious or life-threatening adverse events were documented; thus, it proves that both regimens are safe for this age category. This fact indicates that primaquine is, in general, well tolerated.

Regional variations and considerations

Now that short-course primaquine regimens show superior results globally, considerations for issues such as the prevalence of G6PD deficiency must come into play, and this is still a significant problem in South Asia and Africa, i.e., areas that also have a higher prevalence of malaria. It can be a financial problem to employ G6PD testing for all malaria patients in a resource-limiting setup.

Several reports have supported the implementation of cheap G6PD testing to prevent complications caused by primaquine and to devise better treatment plans [9].

This, therefore, means a more cautious approach plan in the places where there are higher percentages of G6PD deficiency.

Limitations

It is, however, acknowledged that this was a quasi-experimental design with a relatively small sample size and was a single-centre study. Further insight is needed to conduct a multicentric randomised controlled trial and multivariate adjustment for confounders in order to generalise the findings to the general population.

## Conclusions

The high-dose, shorter-course therapy is a promising alternative to the low-dose standard 14-day therapy for the radical cure of vivax malaria. Both regimes have similar efficacy, with a slightly raised incidence of trivial GI symptoms in high-dose therapy; however, significantly improved adherence is seen in this shorter therapy. This implies its consideration in clinical practice, particularly in settings where compliance with extended therapy is problematic.
